# Renal Denervation for Uncontrolled and Resistant Hypertension: Systematic Review and Network Meta-Analysis of Randomized Trials

**DOI:** 10.3390/jcm10040782

**Published:** 2021-02-16

**Authors:** Jonathan Silverwatch, Kristen E. Marti, Mi T. Phan, Hinali Amin, Yuani M. Roman, Vinay Pasupuleti, Maciej Banach, Joshuan J. Barboza, Adrian V. Hernandez

**Affiliations:** 1 School of Pharmacy, University of Connecticut, Storrs, CT 06269, USA; jonathan.silverwatch@uconn.edu (J.S.); kristen.marti@uconn.edu (K.E.M.); mi.phan@uconn.edu (M.T.P.); hinali.amin@uconn.edu (H.A.); yuanniroman@gmail.com (Y.M.R.); 2 MedErgy Health Group Inc., Yardley, PA 19067, USA; lepiscean@gmail.com; 3 Department of Hypertension, Medical University of Lodz, 90-419 Lodz, Poland; maciejbanach@aol.co.uk; 4 Vicerrectorado de Investigación, Universidad San Ignacio de Loyola (USIL), Lima 15024, Peru; jbarbozameca@relaped.com

**Keywords:** renal denervation, resistant hypertension, uncontrolled hypertension, network meta-analysis

## Abstract

Comparative efficacy and safety of renal denervation (RDN) interventions for uncontrolled (UH) and resistant hypertension (RH) is unknown. We assessed the comparative efficacy and safety of existing RDN interventions for UH and RH. Six search engines were searched up to 1 May 2020. Primary outcomes were mean 24-h ambulatory and office systolic blood pressure (SBP). Secondary outcomes were mean 24-h ambulatory and office diastolic blood pressure (DBP), clinical outcomes, and serious adverse events. Frequentist random-effects network meta-analyses were used to evaluate effects of RDN interventions. Twenty randomized controlled trials (RCTs) (*n* = 2152) were included, 15 in RH (*n* = 1544) and five in UH (*n* = 608). Intervention arms included radiofrequency (RF) in main renal artery (MRA) (*n* = 10), RF in MRA and branches (*n* = 4), RF in MRA+ antihypertensive therapy (AHT) (*n* = 5), ultrasound (US) in MRA (*n* = 3), sham (*n* = 8), and AHT (*n* = 9). RF in MRA and branches ranked as the best treatment to reduce 24-h ambulatory, daytime, and nighttime SBP and DBP versus other interventions (p-scores: 0.83 to 0.97); significant blood pressure effects were found versus sham or AHT. RF in MRA+AHT was the best treatment to reduce office SBP and DBP (p-scores: 0.84 and 0.90, respectively). RF in MRA and branches was the most efficacious versus other interventions to reduce 24-h ambulatory SBP and DBP in UH or RH.

## 1. Introduction

Renal denervation (RDN) is an option for treating resistant hypertension (RH) [1] and uncontrolled hypertension (UH) [2]. Early studies such as the Symplicity HTN-1 single-arm trial and the Symplicity HTN-2 randomized controlled trial (RCT) showed promising results of RDN on lowering blood pressure (BP); however, the Symplicity HTN-3 sham-controlled trial in 2014 showed neutral results. Reasons for negative findings include patients’ failure to adhere to antihypertensive medication as well as the inexperience of those performing the renal ablation. More recent RCTs improved the Symplicity HTN-3 trial’s shortcomings; the DENERHTN trial, the SPYRAL HTN-OFF trial, the SPYRAL HTN-ON trial, and the RADIANCE-HTN SOLO trial showed clinically significant decreases in ambulatory BP [3,4].

Several systematic reviews (SR) have evaluated the efficacy of RDN in RH and/or UH [5,6,7,8,9]. Cheng et al. found that UH patients experienced a reduction in mean 24-h systolic blood pressure (SBP) of 4 mmHg (95% confidence interval (CI) −5.5 to −2.6) after RDN when compared to controls [5]. Another recent SR of sham-controlled RCTs by Dahal et al. identified that RDN reduced both ambulatory SBP (−3.5 mmHg, 95% CI −5.0 to −1.9) and diastolic blood pressure (DBP) (−1.9 mmHg, 95% CI −3.6 to −0.2) in both RH and UH patients [6]. Coppolino et al. in 2017 assessed RH patients and found that RDN did not reduce ambulatory and office BP, or clinical outcomes, in comparison to standard therapy or sham [7]. Yao et al., in 2016, found a significant reduction of DBP in comparison to standard medical therapy in RH patients (−3.8 mmHg, 95% CI −7.2 to −0.3) [8]. Finally, Fadl Elmula et al., in 2015, did not find differences in ambulatory or office BPs between RDN + antihypertensives and antihypertensives alone in RH patients [9].

None of the previous systematic reviews compared all available RDN options to one another, in particular specific types of radiofrequency (e.g., main renal artery, main renal artery and branches) or ultrasound. We performed a systematic review and meta-analysis of RCTs of RDN interventions in patients with UH or RH to determine their effects on several intermediate and clinical outcomes. 

## 2. Materials and Methods

### 2.1. Data Sources and Searches

A comprehensive literature search was performed on 1 May 2020 in PubMed, EMBASE, Scopus, Web of Science, the Cochrane library, and clinicaltrials.gov. Keywords were renal denervation, resistant hypertension, uncontrolled hypertension, randomized controlled trials; the PubMed search strategy is available in the Supplemental File.

### 2.2. Study Selection

Abstracts were independently selected by three investigators (JS, VP, JJB). Inclusion criteria were RCTs in ≥18 years-old with RH and/or UH and evaluating RDN interventions such as radiofrequency (RF) in main renal artery (MRA) and branches, RF in MRA, RF in MRA plus antihypertensive therapy (AHT), ultrasound (US) in MRA, sham, and AHT. RCTs with at least one outcome of interest were included. We excluded animal and observational studies. We did not restrict RCTs by sample size, follow-up time, or language. Full texts were reviewed for studies whose eligibility was questioned. Discrepancies were solved by discussion between investigators and a senior investigator (AVH).

Primary outcomes were mean 24-h ambulatory and office SBP. Secondary outcomes were mean 24-h ambulatory and office DBP, daytime SBP and DBP, nighttime SBP and DBP, and the following clinical outcomes: overall mortality, cardiovascular (CV) mortality, stroke, myocardial infarction (MI), hypertensive crisis, heart failure, hospitalization of any cause, renal complications (e.g., doubling of serum creatinine, end stage renal disease), and serious adverse events (SAEs). We used definitions of clinical outcomes as used in the original studies. 

### 2.3. Data Extraction and Quality Assessment

Three investigators (JS, VP, JJB) independently extracted trial information (trial acronym, year of publication, sample size, trial phase, number of RDN interventions, follow-up time, type of patients, type and definition of RDN interventions), patient characteristics (age, body mass index (BMI), proportion of male, races, smokers, history of type 2 diabetes, coronary artery disease (CAD), chronic kidney disease (CKD), obstructive sleep apnea (OSA), number and types of antihypertensives at baseline), and outcome data per trial arm. 

The 2019 Cochrane risk-of-bias (RoB) tool 2.0 was used to assess the risk of bias of RCTs [10]. This evaluated several domains that bias may have risen from: randomization process, deviations from intended interventions (effect of assignment to intervention), missing outcome data, measurement of the outcome, and selection of the reported result. The risk of bias per domain followed an algorithm to conclude the existence of low risk, some concerns, or high risk per domain and per trial. Discrepancies in data extraction or risk of bias assessment were solved by discussion between investigators and a senior investigator (AVH).

### 2.4. Data Synthesis and Analysis

The Preferred Reporting Items for Systematic Reviews and Meta-Analyses Network Meta-Analyses (PRISMA-NMA) guidelines were used to report this systematic review [11].

Inverse variance random-effects meta-analyses were used for all meta-analyses. Effects of RDN on outcomes were expressed as mean differences (MDs) for continuous outcomes, and relative risks (RR) for dichotomous outcomes with their 95% CIs. Best interventions were ranked with the p-score, where best interventions had values closer to one. Statistical heterogeneity of effects among RCTs were evaluated using the I^2^ statistic, with values of <30%, 30–60%, and >60% corresponding to low, medium, and high heterogeneity, respectively. Publication bias was tested with the Egger’s test if more than ten RCTs were available. 

To compare all RDN interventions to one another, we conducted network meta-analyses (NMA) within a frequentist framework [12]. The geometry of the networks per outcome was assessed regarding specific treatments, studies with specific direct comparisons, and individuals randomly assigned to each intervention. RCTs were assessed for similarity or transitivity based on the evaluation of patient characteristics (age, BMI, number of AHT drugs at baseline, type of hypertension) and trial design (sample size, type of RDN intervention, outcomes, follow-up time). NMA comparisons were also assessed for consistency between direct and indirect effects with a test of disagreement for each comparison as well as the Cochran’s Q statistic for the overall network [13]. Sensitivity analyses were performed for trials with a 6-month follow-up time and those only including RH patients. 

When NMAs were not possible due to scarcity of events and/or trial arms, we conducted traditional pairwise meta-analyses between one specific RDN intervention and a given control. We then used the treatment arm continuity correction (TACC) method to correct for zero events in trial arms and the Paule–Mandel method for calculating between-study variance tau^2^. R 3.5.3 software (www.r-project.org, accessed on 20 December 2020) was used for statistical analyses.

## 3. Results

### 3.1. Study Selection

**Figure 1:**
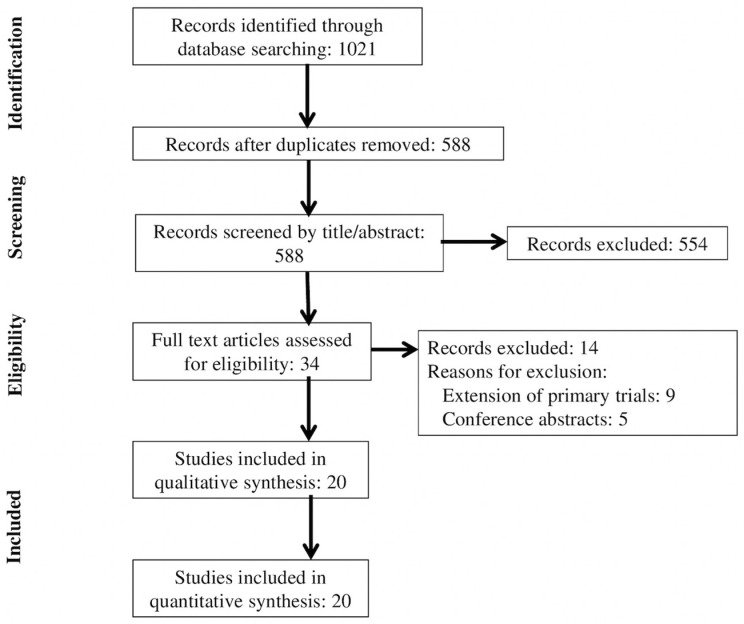
Flowchart of trial selection.

We identified 588 unique articles through a review of titles and abstracts. We assessed 34 full text articles for eligibility, and after review we excluded 14 articles that were extensions of primary trials (*n* = 9) or conference abstracts (*n* = 5) (Figure 1). Twenty RCTs (*n* = 2152) were included in the final analysis [14,15,16,17,18,19,20,21,22,23,24,25,26,27,28,29,30,31,32,33].

### 3.2. Trial Characteristics

Table 1 summarizes key characteristics of RCTs evaluated. Six out of 20 RCTs were performed in multiple countries, and sample sizes ranged from 15 to 535 participants. RH patients were enrolled in 15 studies (*n* = 1544) [16,19,20,21,22,23,25,26,27,28,29,30,31,32,33], and UH patients were enrolled in three studies (*n* = 608) [14,15,17,18,24]. Mean ages ranged from 48 to 64 years-old, mean BMI 27.5 to 34.3 kg/m^2^, and mean number of antihypertensive drugs zero to five. Follow-up time ranged from two to six months, with six months being the majority (*n* = 14).

The most common definition of RH included office SBP ≥140 mmHg and ≥ three antihypertensive medications at maximally tolerated doses, with one being a diuretic. The most common definition of UH included office SBP 150–180 mmHg, mean 24-h ambulatory SBP 140–170 mmHg, and ≤three antihypertensive medications. Intervention arms and number of RCTs with available interventions were the following: RF in MRA and branches (*n* = 4), RF in MRA (*n* = 10), RF in MRA plus AHT (*n* = 5), US in MRA (*n* = 3), sham (*n* = 8), and AHT (*n* = 9). Network geometries for 24-h ambulatory BPs, office BPs, and daytime and nighttime DBP showed direct comparisons for RF MRA vs. antihypertensive or sham as well as for sham vs. US MRA or RF MRA and branches (Figure S1A–D,G,H). For daytime and nighttime SBP, network geometries showed direct comparisons for RF MRA and branches vs. RF MRA vs. US MRA (Figure S1E,F).

### 3.3. Risk of Bias Assessment

Figure S2 summarizes the risk of bias per domain. Most trials had low risk of bias for each domain: randomization process (80%), deviations from intended interventions (89%), missing outcome data (70%), measurement of the outcome (55%), selection of the reported result (80%). Missing outcome data was a high risk of bias in one trial. Overall, there was some concern of bias in the majority of trials (*n* = 12; 60%), which came mostly from measurement of the outcome, missing outcome data, and selection of the reported result. Five trials had low overall risk (25%), and one trial had high overall risk (5%).

### 3.4. Network Meta-Analyses of Primary Outcomes

RF in MRA and branches had a significantly larger reduction in 24-h ambulatory SBP compared to RF in MRA (MD −7.8 mmHg, 95% CI −15.1 to −0.4), to RF in MRA plus AHT (MD −11.9 mmHg, 95% CI −23.4 to −0.4), to sham (MD −7.2 mmHg, 95% CI −13.6 to −0.8), and to AHT (MD −12.9 mmHg, 95% CI −22.6 to −3.2) (Table 2).

For 24-h ambulatory SBP, RF in MRA and branches showed the largest reduction in comparison to other treatments (Figure S3). For office SBP, RF in MRA plus AHT showed the largest reduction in comparison to other treatments; however, 95%CIs were largely overlapping (Table 3, Figure S4).

RF in MRA and branches ranked as the best among other interventions for reducing 24-h ambulatory SBP (p-score = 0.97); AHT ranked as the worst intervention. For office SBP, RF in MRA plus AHT ranked as the best intervention (p-score = 0.84); US MRA ranked as the worst intervention. Consistency of the networks was moderate for 24h ambulatory SBP (*p* = 0.03) and high for office SBP (*p* = 0.74).

### 3.5. Network Meta-Analyses of Secondary Outcomes

RF in MRA and branches had a significantly larger reduction in 24-h ambulatory DBP compared to RF in MRA (MD −4.2 mmHg, 95%CI −8.3 to −0.2), to sham (MD −3.7 mmHg, 95%CI −7.1 to −0.2), and AHT (−6.8 mmHg, 95%CI −12.7 to −0.8) (Table 2). RF in MRA and branches showed the largest reduction in comparison to other treatments for 24-h ambulatory DBP, daytime SBP and DBP, and nighttime SBP and DBP (Figures S5, S7–S10, Tables S1 and S2). For office DBP, RF in MRA plus AHT had a significantly larger reduction compared to AHT (MD −5.4 mmHg, 95%CI −9.6 to −1.1) (Table 3) and showed the largest reduction in comparison to other treatments (Figure S6).

RF in MRA and branches ranked as the best among other interventions for reducing 24-h ambulatory DBP, daytime SBP and DBP, and nighttime SBP and DBP (p-scores: 0.83 to 0.97) (Table S3). AHT ranked as the worst intervention for reducing 24-h ambulatory DBP and nighttime SBP and DBP. RF MRA plus AHT ranked as the worst intervention for reducing daytime SBP and DBP. RF in MRA plus AHT ranked as the best among other interventions for reducing office DBP (p-score = 0.90) (Table S3); sham ranked as the worst intervention. Consistency of the networks was moderate for daytime and nighttime SBP (*p* = 0.07 and 0.08, respectively) and high for other secondary outcomes (*p* range: 0.39 to 0.87).

### 3.6. Sensitivity Analyses

For RCTs with a 6-month follow-up time, rankings of best treatments were similar to main analyses of all primary and secondary outcomes. RF in MRA and branches ranked the highest for 24-h ambulatory, daytime, and nighttime SBP and DBP (p-scores: 0.78 to 0.96), and RF in MRA plus AHT ranked the highest for office SBP and DBP (p-scores: 0.91 and 0.96, respectively). 

For RCTs that included only RH patients, rankings of best treatments were similar to main analyses of 24-h ambulatory and daytime SBP and DBP (RF in MRA and branches p-scores: 0.74 to 0.97) and office SBP and DBP (RF in MRA and AHT p-scores: 0.80 and 0.89, respectively). A difference in rankings was seen in nighttime SBP and DBP, with sham procedure having the highest ranking (p-scores: 0.69 and 0.65, respectively).

### 3.7. Effect of Renal Denervation on Clinical Outcomes

Due to scarcity of clinical outcomes and treatment arms being compared, NMAs were not possible. We then evaluated the effect of some types of RDN interventions (RF MRA, RF MRA and branches, RF MRA plus AHT) vs. controls (sham, AHT) on clinical outcomes in traditional pairwise meta-analyses (Table S4, Figures S11–S26). Clinical outcomes with at least data in two studies were heart failure, stroke, MI, renal complications, hypertensive crisis, and SAEs. None of the effects of RDN interventions on clinical outcomes were significant.

## 4. Discussion

### 4.1. Main Findings

We found that RF in MRA and branches was the best intervention to reduce 24-h ambulatory, and daytime and nighttime SBP and DBP compared to other interventions. Only 24-h ambulatory SBP and DBP were significantly reduced in most of comparisons. RF in MRA plus AHT was the best intervention to reduce office SBP and DBP compared to other interventions, but neither effect was significant. Best RDN interventions were similar after analyses in 6-month follow-up and RH only trials. We did not find any significant effect of RDN interventions on scarce clinical outcome data. Most trials had overall low risk or some concern of bias.

### 4.2. What Is Known in the Literature

RH is estimated to affect 10% of individuals with hypertension [34]; 54% of individuals with hypertension are uncontrolled [2]. Among individuals with UH, 39.4% were unaware of their hypertension, 15.8% were aware but not on any medication, and 44.8% were aware and not at maximally tolerated doses [2].

RDN procedures most commonly use either RF or US energy. The first-generation RF ablation catheter, Symplicity Flex^®^ (Medtronic, Minneapolis, MN, USA), was used in several RDN RCTs [20,21,23,25,26,27,28,29,30,31,32,33]. The second-generation RF ablation catheter, Symplicity Spyral^®^ (Medtronic), was used in more recent RCTs [16,18,24]. The Paradise US catheter^®^ (ReCor Medical, Palo Alto, CA, USA) has been used in two recent RCTs [16,17].

The efficacy of RDN has been previously evaluated in several SRs (Table S5) [5,6,7,8,9]. Coppolino et al., Yao et al., and Fadl Elmula et al. included only RH patients [7,8,9]. Cheng et al. included only UH patients [5], and Dahal et al. included patients with both RH and UH [6]. All SRs included between 985 and 1539 individuals. All except Fadl Elmula et al. assessed risk of bias with the 2011 Cochrane tool. Both Fadl Elmula et al. and Coppolino et al. concluded that RDN did not have a significant effect on BP vs. no intervention and standard treatment or sham, respectively. Yao et al. concluded that RF RDN was not superior to standard treatment. Both Cheng et al. and Dahal et al. concluded that RDN significantly reduced 24-h ambulatory SBP vs. any control and sham, respectively. Additionally, Cheng et al. found a significant reduction in office SBP, and Dahal et al. found a significant reduction in 24-h ambulatory and office DBP.

Coppolino et al. and Cheng et al. assessed the effect of RDN on clinical outcomes. Coppolino et al. showed low quality evidence that RDN did not affect major CV events or renal function but did increase bradycardia events. Cheng et al. concluded that RDN did not increase major adverse events (Table S5). Several meta-analyses have shown that BP reduction is associated with a lower incidence of CV events. The HOPE-3 trial showed that patients with an SBP reduction of −5.8/−3.0 mmHg (baseline office SBP > 143.5 mmHg) experienced 28% fewer CV events when treated with AHT vs. placebo [35]. CV outcomes have not been evaluated as primary outcomes in any RDN trials to date, but it is estimated that a 10-mmHg reduction in office SBP maintained after RDN among individuals with an average age of 65 years-old would be associated with a 25% lower incidence of CV events [3].

Mahfoud et al. conducted a single-arm trial of alcohol-mediated renal denervation using the Peregrine Catheter in patients with hypertension, which showed promising results in patients with UH. The ambulatory and office BPs were significantly reduced by −11/−7 mm Hg, respectively, after 6 months. Within 1 month of procedure, there was no report of any major adverse events and death in 95% of patients. Further studies or RCTs are needed to confirm the safety and efficacy of the procedure and compare them to other available treatments [36]. 

### 4.3. What Our Study Adds to the Literature

We performed an NMA of all available and most updated RDN interventions, which no previous SR has performed. Our study evaluated newer RDN interventions such as RF MRA and branches and US MRA. We found that RF MRA and branches consistently provided the largest reduction in 24-h ambulatory, daytime and nighttime SBP and DBP. The Symplicity Spyral was specifically designed to also treat RA branches with a diameter of 3 to 8 mm [4]. Having a higher number of ablated sites may improve the chance of hitting pressor nerves around the arteries [3]. This concept is also supported by the moderate effect achieved by the US intervention [17].

We used follow-up times specified in the primary outcomes, even in cases when the RCTs were followed up beyond that time point. We also performed sensitivity analyses by removing studies with follow-up shorter than six months and found results consistent with main analyses. Eight different BP outcomes were evaluated, including both in-office and out-of-office measurements. Ambulatory BP monitoring is regarded as a more accurate BP measuring option as it avoids the “white coat effect” of office point-of-care BP measures [37].

We found data from two to five RCTs for heart failure, stroke, MI, renal complications, and hypertensive crisis. Additionally, SAEs were available in two to three RCTs. NMAs were not possible though. Our pairwise meta-analyses comparing RF MRA +/− branches to sham did not find significant effects on any of the clinical outcomes. Other clinical outcomes such as overall mortality, CV mortality, and hospitalizations of any cause were available for one or none of the RCTs. Pairwise meta-analyses comparing RF MRA +/− branches or AHT to sham or AHT did not show significant differences for SAEs. We will need more clinical outcome data in future trials to elucidate the effects of RDN interventions on harder outcomes.

Our study also ranked RDN interventions per outcome by using p-scores in the context of NMA. P-scores are equivalent to the surface under the cumulative ranking curve (SUCRA) in Bayesian NMA [38]. Finally, we used the new 2019 Cochrane risk-of-bias tool 2.0 to evaluate the risk of bias for each RCT. In contrast to the old 2011 version, items have been grouped in domains of bias, decisions per domain include the option of some concern of bias instead of “unclear risk of bias”, and documentation is more detailed.

### 4.4. Limitations

First, we identified heterogeneity among included RCTs in terms of age, sex, BMI, type of population, and follow-up time. To adjust for heterogeneity, we performed sensitivity analyses on RCTs including only RH patients and only trials with a 6-month follow-up time. For most outcomes, results were consistent with the main analyses. Second, the majority of included RCTs had some concern of bias, coming mainly from measurement of the outcome, missing outcome data, and selection of the reported result; one RCT of 81 RH patients had a high risk of bias because of missing outcome data [19]. Third, the mean number of AHT drugs varied among 15 RH RCTs, ranging from three to five; this may change the baseline risk of patients undergoing RDN interventions. Lastly, there was scarce data available to analyze clinical outcomes, so we could not conduct NMA on these outcomes. Instead, we performed traditional pairwise meta-analyses between one specific RDN intervention and a given control. Data for overall mortality, CV mortality, and hospitalizations were very scarce, in line with previous SRs [5,6,7,8,9].

## 5. Conclusions

Our systematic review and network meta-analyses found that RF in MRA and branches was the most efficacious RDN intervention compared to other interventions to reduce BP outcomes in RH or UH populations. No significant difference in the effect of RDN on clinical outcomes was found, but data were scarce and outcomes were uncommonly described in existing trials. More clinical outcome data is needed from future trials to further assess the efficacy and safety of RDN interventions.

## Figures and Tables

**Table 1:** Baseline characteristics of included randomized controlled trials.

Acronym/Author, Year [reference]	Country (ies)	RDN Arm(s); Sample	Follow-Up Time (mo)	Type of HTN Patients; Definition of HTN	Description of RDN Type (s)	Type of Control Arm; Description of Control	Primary Outcome	Mean Age (SD)	Mean BMI (SD)	Number of AHT Drugs Mean (SD)
REDUCE HTN: REINFORCE/Weber, 2020 [14]	USA	RF MRA; 51	2	UH; office SBP of ≥150 and <180 mm Hg, an average 24-h ambulatory SBP of ≥135, and <170 mm Hg after 4-week antihypertensive medication washout	RF of the full renal artery length	Sham; renal angiography alone	Mean reduction in ambulatory SBP at 2 months	58.4 (9.9)	NR	Off AHT
SPYRAL Pivotal/Bohm, 2020 [15]	Australia, Austria, Canada, Germany, Greece, Ireland, Japan, the UK, and the USA	RF MRA + branches; 331	3	UH; office SBP 150 to 180 mmHg, office DBP at least 90 mmHg	The catheter has four electrodes designed to simultaneously or individually deliver radiofrequency ablation (intended duration of 60 s) to all four quadrants of the renal arteries and branch vessels with each activation; 45 s or longer was considered a successful ablation	Sham; renal angiogram only	Mean reduction in ambulatory SBP at 3 months	52.5 (10.6)	31.0 (5.7)	Off AHT
RADIOSOUND HTN/Fengler, 2019 [16]	Germany	US MRA; 120	3	RH; office SBP >160 or DBP >90 despite treatment with ≥3 classes of AHT drugs on ≥50% max doses including ≥1 diuretic. AHT meds had to be stable for ≥4 weeks	US Paradise catheter; balloon cooled devise creates fully circumferential thermal ablation using acoustic energy	RF MRA; RF MRA + branches; RF: Symplicity Spyral catheter; administers ≤ 4 ablations simultaneously in spiral pattern using heat	Change in ambulatory daytime SBP at 3 months	63.5 (9.4)	31.6 (5.6)	5.0 (1.4)
RADIANCE HTN SOLO/Azizi, 2018 [17]	USA, Germany, Netherlands, Belgium, UK	US MRA; 146	2	UH; at screening: uncontrolled office BP ≥140/90 but <180/110 on 0 to 2 drugs; or controlled: office BP <140/90 on 1 to 2 drugs. After 4 weeks of AHT discontinuation: Ambulatory SBP ≥135, DBP ≥85 and <170/105	US Paradise RDN system; patients received about 5.4 US emissions in the MRAs; 9 patients received ablation in accessory RAs; ≥2 US emissions were performed in 71 patients	Sham; renal angiogram only	Change in ambulatory daytime SBP at 2 months	53.9 (10.1)	29.5 (5.4)	Off AHT
SPYRAL HTN ON/Kandazari, 2018 [18]	USA, Germany, Japan, UK, Australia, Austria, Greece	RF MRA + branches; 80	6	UH; office SBP 150 to 180 and DBP ≥90. 24-h ambulatory BP 140 to 170 at second screening and were on 1 to 3 AHT meds ≥50% of the max doses for ≥6 weeks	Symplicity Spyral catheter; circumferential RF ablation in the 4 quadrants of the RA and branch vessels between 3 and 8 mm in diameter	Sham; renal angiogram required to stay on the procedure table for at least 20 min with sensory masking post angiogram	Ambulatory BP change at 6 months	53.4 (9.8)	32 (5.5)	2.3 (0.85)
WAVE IV/Schmeider, 2018 [19]	Czech Republic, Germany, New Zealand, Poland, UK	US MRA; 81	6	RH; office SBP ≥160 while taking ≥3 AHT meds at max tolerated doses with 1 being a diuretic	Kona Surround Sound system; bilateral RDN using therapeutic levels of ultrasound energy	Sham; bilateral sham treatment using diagnostic levels of ultrasound energies	Difference in office SBP at 24 weeks	61.1 (11.1)	29.8 (4.3)	4.6 (1.5)
Warchol-Celinska, 2018 [20]	Poland	RF MRA; 60	3	RH + OSA; office SBP ≥140, mean daytime SBP ABPM ≥135 mmHg receiving and adhering to full doses of ≥3 AHT drugs including a diuretic for ≥4 weeks before screening	Symplicity Flex catheter; RF ablations of ≤8 W were applied, lasting <2 min each to obtain ≤6 ablations separated both longitudinally and rotationally within RA	AHT; maintenance of AHT medications only	Reduction in office SBP at 3 months	55.2 (9.2)	34.3 (5.4)	4.95 (0.87)
SYMPATHY/De Jager, 2017 [21]	Netherlands	RF MRA + AHT; 139	6	RH; average daytime SBP ABPM ≥135 despite use of ≥3 BP lowering agents	Symplicity Flex catheter (*n* = 60); EnligHTN ablation catheter (*n* = 40). Mean number of ablations was 15; also, usual AHT	AHT; medication only	Change in daytime systolic ABPM after 6 months	61.4 (11.4)	28.8 (4.7)	3.4 (1.3)
INSPIRED/Jacobs, 2017 [22]	Belgium	RF MRA + AHT; 15	6	RH; 24-h ambulatory SBP ≥130 or DBP ≥80 while taking ≥3 AHT meds	EnligHTN catheter; RDN + usual AHT; 8 to 12 ablations were made from the distal MRA to the ostium. In case of a long RA, the basket was placed more proximal	AHT; optimized drug regimen maintenance	Change in 24-h ambulatory SBP and in glomerular filtration rate at 6 months	48.1 (9.3)	30.3 (4.6)	4.0 (1.5)
Pekarskiy, 2017 [23]	Russia	RF MRA + branches; 51	6	RH; office SBP ≥160 or DBP ≥100 despite stable (>3 mo) prescribed treatment with full doses of ≥3 AHT drugs including a diuretic	Symplicity Flex catheter; further advanced into segmental branches beyond main bifurcation, 2–4 separate point treatments per branch; 4 lesions if branch diameter ≥4 mm, 2 lesions if less	RF MRA; Symplicity Flex catheter; ablations in MRA only	Change in 24-h mean ambulatory SBP at 6 months	55.7 (8.6)	NR	4.1 (0.9)
SPYRAL HTN OFF/Townsend, 2017 [24]	USA, Germany, Japan, UK, Australia, Austria, Greece	RF MRA + branches; 80	3	UH; office SBP 150 to 180, DBP ≥90, and a mean 24-h ambulatory SBP 140 to 170	Symplicity Spyral catheter; 4 electrodes positioned to apply RF energy circumferentially in all 4 quadrants of the RA and branch vessels	Sham; renal angiogram only	BP reduction based on ABPM at 3 months	54.2 (10.9)	30.0 (5.1)	Off AHT
ReSET/Mathiassen, 2016 [25]	Denmark	RF MRA; 69	6	RH; daytime ABPM SBP ≥ 145, one month of stable AHT with ≥3 meds including a diuretic	Symplicity Flex catheter; 4–6 RF treatments of 5–8 W were applied for 2 min to cover the entire circumference in a spiral manner along the length of each MRA	Sham; dummy radiograph scan performed for another 10–15 min before removing sheath	Change in daytime ABPM SBP at 3 months	55.7 (8.8)	28.5 (4.5)	4.1 (1.1)
DENERVHT/Oliveras, 2016 [26]	Spain	RF MRA + AHT; 24	6	RH; office SBP ≥150 and 24-h SBP ≥140 despite getting ≥3 full dose AHT drugs (1 a diuretic but no aldosterone antagonist)	Symplicity Flex catheter; 4–6 applications of low power RF energy (8 W) delivered to each RA from distal to proximal; also, baseline AHT	AHT; baseline AHT + 25 mg spironolactone with forced titration to 50 mg after 1 mo	Change in ambulatory 24-h SBP at 6 months	63.5 (7.5)	32.0 (5.7)	4.1 (0.7)
DENERHTN/Azizi, 2015 [27]	France	RF MRA + AHT; 106	6	RH; office SBP ≥140 or DBP ≥90 despite stable regimen of max doses of ≥3 AHT drugs of different classes, including a diuretic	Symplicity Flex catheter; 2–4 weeks after randomization	AHT; spironolactone 25 mg/d, bisoprolol 10 mg/d, SR prazosin 5 mg/d, rilmenidine 1 mg/d added to triple drugs	Change in daytime ambulatory SBP at 6 months	55.2 (10.4)	30.2 (4.7)	NR
Desch, 2015 [28]	Germany	RF MRA; 71	6	RH; mean daytime SBP on 24-h ambulatory BP measurement 135 to 149 or DBP 90 to 94 despite intake of ≥3 AHT drugs including a diuretic at max tolerated doses	Symplicity Flex catheter; 4–6 ablation runs for 2 min in each RA delivered circumferentially to wall from distal to proximal	Sham; saline infusion, angiography of renal arteries and simulated RDN procedure with 4–6 sham runs	Change in 24-h SBP at 6 months	60.9 (8.8)	31.5 (4.5)	4.3 (1.3)
SYMPLICITY HTN JAPAN/Kario, 2015 [29]	Japan	RF MRA; 41	6	RH; office SBP ≥160 on stable regimen of ≥3 AHT classes at max tolerated dose including a diuretic for ≥6 w prior to enrollment. 24-h ambulatory SBP ≥135	Symplicity Flex catheter; 4–6 ablations in each RA in a helical pattern, rotating as the catheter is pulled back from the distal portion to the proximal portion of the MRA	AHT; current AHT only	Change in office SBP at 6 months	57.9 (12.4)	27.5 (4.8)	4.9 (1.6)
PRAGUE-15/Rosa, 2015 [30]	Czech Republic	RF MRA; 106	6	RH; office SBP >140 after treatment with ≥3 AHT drugs at optimal doses, including a diuretic	Symplicity Flex catheter; 4–6 applications of low power RF energy to each RA. Helical fashion within the artery by rotating the catheter during pullback. Distance between ablation sites was 5 mm	AHT; including spironolactone	SBP and DBP by 24-h ABPM at 6 months	57.5 (10.6)	32.3 (4.6)	5.2 (1.2)
SYMPLICITY HTN-3/Bhatt, 2014 [31]	USA	RF MRA; 535	6	RH; SBP of ≥160 and taking max tolerated doses of ≥3 AHT drugs of complementary classes, 1 being a diuretic	Symplicity Flex catheter; delivers low-level RF energy (8W); 4 to 6 ablations up to 120 s to the distal RA and rotating helical pattern	Sham; renal angiography only	Change in office SBP at 6 months	57.4 (10.7)	34.1 (6.5)	5.1 (1.4)
OSLO RDN/Fadl Elmula, 2014 [32]	Norway	RF MRA; 20	6	RH; office SBP >140 despite max tolerated doses of ≥3 AHT drugs including a diuretic	Symplicity Flex catheter system as described in SYMPLICITY HTN-2 trial	AHT; AHT medication adjusted at baseline, 1 and 3 months	Change in office SBP at 6 months	59.8 (8.8)	29.5 (5.2)	5.0 (1.4)
SYMPLICITY HTN-2/Elser, 2010 [33]	Europe, Australia, New Zealand	RF MRA + AHT; 106	6	RH; SBP ≥160 despite compliance with ≥3 AHT drugs	Symplicity Flex catheter; 4–6 discrete low power RF treatments applied along the length of both MRA	AHT; maintenance of previous AHT	Change in seated office SBP at 6 months	58 (12)	31 (5)	5.2 (1.6)

All blood pressures measured as mmHg. HTN: hypertension; BMI: body mass index; US: ultrasound; MRA: main renal artery; RDN: renal denervation; SBP: systolic blood pressure; DBP: diastolic blood pressure; SD: standard deviation; IQR: interquartile range; AHT: antihypertensive; RF: radio frequency; OSA: obstructive sleep apnea; ABPM: ambulatory blood pressure monitoring; NR: not reported; RH: resistant hypertension; UH: uncontrolled hypertension.

**Table 2:** League table of the effects of treatments expressed as MD and their 95%CIs on 24-h ambulatory systolic blood pressure (white cells) and 24 h ambulatory diastolic blood pressure (gray cells).

RF MRA + branches	**−4.2 (−8.3 to −0.2)**	−6.6 (−13.4 to 0.25)	−2.5 (−7.8 to 3.7)	**−3.7 (−7.1 to −0.2)**	**−6.8 (−12.7 to −0.8)**
**−7.8 (−15.1 to −0.4)**	RF MRA	−2.3 (−7.8 to 3.1)	1.7 (−3.0 to 6.4)	0.6 (−2.1 to 3.2)	−2.5 (−6.8 to 1.8)
**−11.9 (−23.4 to −0.4)**	−4.1 (−13.0 to 4.8)	RF MRA + AHT R	4.0 (−3.2 to 11.2)	2.9 (−3.2 to 8.9)	−0.2 (−3.5 to 3.1)
−6.0 (−15.8 to 3.8)	1.8 (−7.1 to 10.7)	5.9 (−6.7 to 18.5)	US MRA	−1.1 (−5.1 to 2.8)	−4.2 (−10.6 to 2.2)
**−7.2 (−13.6 to −0.8)**	0.6 (−4.4 to 5.5)	4.7 (−5.5 to 14.8)	−1.2 (−8.6 to 6.2)	Sham S	−3.1 (−8.1 to 1.2)
**−12.9 (−22.6 to −3.2)**	5.9 (−11.4 to 1.3)	−1.0 (−7.2 to 5.2)	−6.9 (−17.8 to 4.1)	−5.6 (−13.7 to 2.4)	AHT

For ambulatory systolic blood pressure, the comparison is column vs. row (comparator); for ambulatory diastolic blood pressure the comparison is row vs. column (comparator). Effects in bold are statistically significant. MD: mean difference; CI: confidence interval; RF: radio frequency; MRA: main renal artery; US: ultrasound; AHT: antihypertensive therapy.

**Table 3:** League table of the effects of treatments expressed as MD and their 95%CIs on office systolic blood pressure (white cells) and office diastolic blood pressure (gray cells).

RF MRA + branches	−2.2 (−10.6 to 6.3)	3.2 (−6.3 to 12.7)	−3.1 (−10.9 to 4.7)	−3.5 (−8.6 to 1.5)	−2.2 (−10.6 to 6.3)
−6.7 (−22.2 to 8.9)	RF MRA	6.1 (−0.9 to 13.0)	−0.2 (−8.2 to 7.8)	−0.7 (−6.0 to 4.6)	0.7 (−4.76 to 6.2)
3.64 (−18.1 to 25.4)	10.3 (−4.9 to 25.5)	RF MRA + AHT	−6.3 (−16.9 to 4.3)	−6.7 (−15.5 to 2.0)	**−5.4 (−9.6 to −1.1)**
−9.15 (−29.2 to 10.9)	−2.46 (−22.6 to 17.7)	−12.8 (−38.0 to 12.5)	US MRA	−0.5 (−6.4 to 5.5)	0.9 (−8.8 to 10.6)
−6.9 (−19.9 to 6.3)	−0.2 (−13.4 to 13.1)	−10.5 (−30.7 to 9.7)	2.3 (−12.9 to 17.5)	Sham	1.4 (−6.2 to 9.0)
−7.3 (−26.4 to 11.8)	−0.7 (−11.7 to 10.4)	**−10.1 (−21.4 to −0.6)**	1.8 (−21.2 to 24.8)	−0.5 (−17.7 to 16.7)	AHT

For office systolic blood pressure, the comparison is column vs. row (comparator); for office diastolic blood pressure the comparison is row vs. column (comparator). Effects in bold are statistically significant. MD: mean difference; CI: confidence interval; RF: radio frequency; MRA: main renal artery; US: ultrasound; AHT: antihypertensive therapy.

## Supplementary Materials

The following are available online https://www.mdpi.com/2077-0383/10/4/782/s1, Supplemental Methods: PubMed search strategy; Table S1: League table of the effects of treatments expressed as MD and their 95%CIs on daytime systolic blood pressure (white cells) and daytime diastolic blood pressure (gray cells); Table S2: League table of the effects of treatments expressed as MD and their 95%CIs on nighttime systolic blood pressure (white cells) and nighttime diastolic blood pressure (gray cells); Table S3: Ranking of renal denervation treatments per outcome; Table S4: Meta-analyses of clinical outcomes; Table S5: Characteristics of previously published systematic reviews compared to our study; Figure S1: Network geometries for primary and secondary outcomes; Figure S2: Risk of bias per domain of included randomized trials; Figure S3: Effect of renal denervation interventions on change of 24 h ambulatory SBP in comparison to antihypertensive drugs; Figure S4: Effect of renal denervation interventions on change of office SBP in comparison to antihypertensive drugs; Figure S5: Effect of renal denervation interventions on change of 24 h ambulatory DBP in comparison to antihypertensive drugs; Figure S6: Effect of renal denervation interventions on change of office DBP in comparison to antihypertensive drugs; Figure S7: Effect of renal denervation interventions on change of daytime SBP in comparison to antihypertensive drugs; Figure S8: Effect of renal denervation interventions on change of nighttime SBP in comparison to antihypertensive drugs; Figure S9: Effect of renal denervation interventions on change of daytime DBP in comparison to antihypertensive drugs; Figure S10: Effect of renal denervation interventions on change of nighttime DBP in comparison to antihypertensive drugs; Figure S11: Effect of RF MRA vs. sham on heart failure; Figure S12: Effect of RF MRA vs. sham on stroke; Figure S13: Effect of RF MRA + branch vs. sham on stroke; Figure S14: Effect of RF MRA +/− branch vs. sham on stroke; Figure S15: Effect of RF MRA vs. sham on myocardial infarction; Figure S16: Effect of RF MRA + branch vs. sham on myocardial infarction; Figure S17: Effect of RF MRA +/− branch vs. sham on myocardial infarction; Figure S18: Effect of RF MRA vs. sham on renal complications; Figure S19: Effect of RF MRA + branch vs. sham on renal complications; Figure S20: Effect of RF MRA +/− branch vs. sham on renal complications; Figure S21: Effect of RF MRA vs. sham on hypertensive crisis; Figure S22: Effect of RF MRA + branch vs. sham on hypertensive crisis; Figure S23: Effect of RF MRA +/− branch vs. sham on hypertensive crisis; Figure S24: Effect of RF MRA vs. sham on serious adverse events; Figure S25: Effect of RF MRA + AHT vs. AHT on serious adverse events; Figure S26: Effect of RF MRA +/− branch vs. sham on serious adverse events.
