# Supplementary material for: Renal Denervation for Uncontrolled and Resistant Hypertension: Systematic Review and Network Meta-Analysis of Randomized Trials
Source: J Clin Med. 2021 Feb 16;10(4):782. doi: 10.3390/jcm10040782 (PMC7919811; doi:10.3390/jcm10040782)

Renal denervation for uncontrolled and resistant hypertension: a systematic review and network meta-analysis of randomized controlled trials.

Jonathan Silverwatch, Kristen E. Marti, Mi T. Phan, Hinali Amin, Yuani M. Roman, Vinay Pasupuleti, Maciej Banach, Joshuan J. Barboza, Adrian V. Hernandez

Supplemental Methods: PubMed search strategy

(renal[All Fields] AND ("denervation"[MeSH Terms] OR "denervation"[All Fields])) AND ((resistant[All Fields] AND ("hypertension"[MeSH Terms] OR "hypertension"[All Fields])) OR (uncontrolled[All Fields] AND ("hypertension"[MeSH Terms] OR "hypertension"[All Fields]))) AND (("randomized controlled trial"[Publication Type] OR "randomized controlled trials as topic"[MeSH Terms] OR "randomized controlled trials"[All Fields] OR "randomised controlled trials"[All Fields]) OR ("randomized controlled trial"[Publication Type] OR "randomized controlled trials as topic"[MeSH Terms] OR "randomised controlled trials"[All Fields] OR "randomized controlled trials"[All Fields]) OR (("random allocation"[MeSH Terms] OR ("random"[All Fields] AND "allocation"[All Fields]) OR "random allocation"[All Fields] OR "randomized"[All Fields]) AND ("Trials"[Journal] OR "trials"[All Fields])) OR (randomised[All Fields] AND ("Trials"[Journal] OR "trials"[All Fields])))

Table S1. League Table of the effects of treatments expressed as MD and their 95%CIs on daytime systolic blood pressure (white cells) and daytime diastolic blood pressure (gray cells). For daytime systolic blood pressure the comparison is column vs row (comparator); for daytime diastolic blood pressure the comparison is row vs column (comparator). Effects in bold are statistically significant.

RF MRA + branches	-5.2 (-10.6 to 0.2)	-7.6 (-15.7 to 0.5)	-1.4 (-9.5 to 6.6)	-4.0 (-8.5 to 0.5)	-6.7 (-13.6 to 0.3)
-5.7 (-12.9 to 1.5)	RF MRA	-2.4 (-8.4 to 3.6)	3.8 (-4.0 to 11.5)	1.2 (-2.3 to 5.1)	-1.5 (-5.9 to 2.9)
-7.7 (-20.2 to 4.8)	-7.7 (-20.2 to 4.8)	RF MRA + AHT	6.2 (-3.6 to 16.0)	3.6 (-3.6 to 10.7)	0.9 (-3.2 to 5.0)
-0.1 (-8.6 to 8.4)	5.6 (-2.6 to 13.8)	7.6 (-5.5 to 20.7)	US MRA	-2.6 (-9.3 to 4.1)	-5.2 (-14.1 to 3.7)
-4.8 (-11.3 to 1.9)	-1.0 (-5.1 to 7.0)	2.9 (-8.9 to 14.8)	-4.7 (-11.8 to 2.5)	Sham	2.6 (-4.1 to 9.3)
-6.9 (-17.2 to 3.3)	-1.2 (-8.5 to 6.1)	0.8 (-6.4 to 7.8)	-6.9 (-17.9 to 4.2)	-2.2 (-11.7 to 7.3)	AHT

MD: mean difference; CI: Confidence interval; RF: Radiofrequency. MRA: Main renal artery, US: Ultrasound, AHT: Antihypertensive therapy.

Table S2. League Table of the effects of treatments expressed as MD and their 95%CIs on nighttime systolic blood pressure (white cells) and nighttime diastolic blood pressure (gray cells). For nighttime systolic blood pressure the comparison is column vs row (comparator); for nighttime diastolic blood pressure the comparison is row vs column (comparator). Effects in bold are statistically significant.

RF MRA + branches	-5.4 (-11.5 to 0.1)	-4.0 (-13.5 to 5.4)	-3.2 (-12.2 to 5.7)	-3.9 (-9.0 to 1.1)	-7.0 (-15.2 to 1.4)
**-7.6 (-14.6 to -0.7)**	RF MRA	1.3 (-5.8 to 8.5)	2.1 (-6.5 to 10.7)	1.4 (-3.0 to 5.9)	-1.6 (-7.2 to 4.1)
-7.3 (-20.1 to 5.6)	0.3 (-10.5 to 11.1)	RF MRA + AHT	-1.3 (-8.5 to 5.8)	0.1 (-8.4 to 8.6)	-2.9 (-7.3 to 1.5)
-2.3 (-10.9 to 5.3)	4.9 (-3.0 to 12.7)	-0.3 (-11.1 to 10.5)	US MRA	-0.7 (-8.1 to 6.7)	-3.7 (-14.0 to 6.6)
-4.7 (-11.0 to 1.5)	2.9 (-2.9 to 8.9)	2.6 (-9.7 to 14.8)	-2.0 (-8.7 to 4.7)	Sham	-3.0 (-10.2 to 4.2)
-10.4 (-21.3 to 0.6)	-2.7 (-11.1 to 5.7)	-3.1 (-9.8 to 3.7)	-7.6 (-19.1 to 3.9)	-5.6 (-15.8 to 4.6)	AHT

MD: Mean difference; CI: Confidence interval; RF: Radiofrequency. MRA: Main renal artery, US: Ultrasound, AHT: Antihypertensive therapy.

Table S3. Ranking of renal denervation treatments per outcome. A higher p-score means such a treatment ranks better than others with lower p-scores for a given outcome.

Treatment arms	Outcomes							
	24h ambulatory SBP	24h ambulatory DBP	Office SBP	Office DBP	Daytime SBP	Daytime DBP	Nighttime SBP	Nighttime DBP
**RF MRA + branches**	0.97	0.95	0.72	0.69	0.83	0.90	0.90	0.88
**RF MRA**	0.51	0.45	0.39	0.35	0.36	0.40	0.30	0.34
**RF MRA + AHT**	0.24	0.22	0.84	0.90	0.26	0.17	0.41	0.54
**US MRA**	0.62	0.68	0.31	0.36	0.81	0.75	0.70	0.56
**Sham**	0.54	0.53	0.39	0.27	0.43	0.52	0.54	0.50
**AHT**	0.12	0.16	0.35	0.43	0.30	0.26	0.14	0.18

SBP: Systolic blood pressure; DBP: Diastolic blood pressure; RF: Radiofrequency; MRA: Main renal artery; AHT: Antihypertensive therapy; US: Ultrasound; NA: Not available.

Table S4. Meta-analyses of clinical outcomes.

Outcome	Number of studies	Intervention	Control	RR (95%CI)	I^2
Heart Failure	2	RF MRA	Sham	1.40 (0.41 to 4.80)	0%
Stroke	3	RF MRA	Sham	0.80 (0.22 to 3.00)	0%
	2	RF MRA + branch	Sham	0.52 (0.04 to 6.13)	0%
	5	RF MRA (+/- branch)	Sham	0.73 (0.23 to 2.33)	0%
Myocardial Infarction	2	RF MRA	Sham	0.98 (0.28 to 3.36)	0%
	2	RF MRA + branch	Sham	1.00 (0.06 to 15.77)	0%
	4	RF MRA (+/- branch)	Sham	0.98 (0.32 to 3.03)	0%
Renal Complications	2	RF MRA	Sham	2.62 (0.51 to 13.56)	0%
	2	RF MRA + branch	Sham	1.00 (0.06 to 15.84)	0%
	4	RF MRA (+/- branch)	Sham	2.03 (0.49 to 8.36)	0%
Hypertensive Crisis	2	RF MRA	Sham	0.55 (0.23 to 1.30)	0%
	2	RF MRA + branch	Sham	1.93 (0.16 to 22.84)	0%
	4	RF MRA (+/- branch)	Sham	0.63 (0.28 to 1.42)	0%
Serious Adverse Events	2	RF MRA	Sham	1.69 (0.31 to 9.17)	0%
	2	RF MRA + AHT	AHT	1.36 (0.59 to 3.11)	53%
	3	RF MRA (+/- branch)	Sham	1.56 (0.33 to 7.35)	0%

RF: radiofrequency; MRA: main renal artery; AHT: antihypertensive therapy

Table S5. Characteristics of previously published systematic reviews compared to our study.

**Characteristics**	**Fadl Elmula et al. 2015**	**Yao et al. 2016**	**Coppolino et al. 2017**	**Dahal et al. 2019**	**Cheng et al. 2019**	**Our study**
Type of review	Systematic review and meta-analysis	Systematic review and meta-analysis	Systematic review and meta-analysis	Systematic review and meta-analysis	Systematic review and meta-analysis	Systematic review and network meta-analysis
Primary objectives	To sum up the randomized evidence on the efficacy and safety of RDN as treatment modality in treatment-resistant hypertensive patients.	To evaluate the efficiency of RDN on RH.	To evaluate the short- and long-term effects of RDN in RH on clinical endpoints and potential adverse events related to the procedure.	To evaluate the efficacy and utility of RDN procedure in RH and UH.	To assess the efficacy and safety of RDN for the treatment of UH.	To assess the comparative efficacy and safety of existing RDN interventions for UH and RH.
Inclusion criteria	RCTs comparing RDN vs no intervention in RH patients on unchanged or optimized AHT (≥3 drug classes, SBP ≥140, 135, or 130 on office, daytime, or 24-h ABPM).	RCTs comparing RDN vs standard medical therapy (≥3 AHT drugs including a diuretic) in RH patients.	RCTs that compared RDN to standard therapy or sham procedure to treat RH, without language restriction.	Sham controlled trials comparing outcomes of RDN in adults with hypertension reporting one of the following: change in 24-h ambulatory BP, office BP, or daytime and nighttime ambulatory BP.	RCTs including a study protocol and evaluating participants randomly allocated to RDN or control. SBP of at least 140, 135, or 130 office, daytime or 24-h ambulatory measurements respectively. Sample size of at least 40.	RCTs in >18 years-old, with RH and/or UH evaluating RDN interventions: RF in MRA and branches, RF in MRA, RF in MRA plus AHT, US in MRA, sham, and AHT.
Databases searched	PubMed, EMBASE, clinicaltrials.gov	PubMed, EMBASE, Cochrane Central	The Cochrane Hypertension Group Specialised Register, CENTRAL, MEDLINE, EMBASE, clinicaltrials.gov	PubMed, EMBASE, CINAHL, Cochrane Central Register of Clinical Trials	Medline, Cochrane library, EMBASE	PubMed, EMBASE, Scopus, Web of Science, the Cochrane library, clinicaltrials.gov
Years of study publication searched	January 2009 to unspecified month in 2015	Up to May 2015	Up to February 2016	Up to September 2018	January 2009 to July 2018	Up to May 2020
Number of studies included	7 RCTs	9 RCTs	12 RCTs	7 RCTs	12 RCTs	20 RCTs

Sample size	985 randomized (958 analyzed)	1059 randomized (988 analyzed)	1149 randomized (sample analyzed varied according to outcome)	1098 randomized (1055/1047 analyzed for safety and efficacy)	1539 randomized (unspecified sample analyzed)	2152 randomized (sample analyzed varied according to outcome)
Risk of bias assessment	No assessment	2011 Cochrane risk of bias tool	2011 Cochrane risk of bias tool	2011 Cochrane risk of bias tool	2011 Cochrane risk of bias tool	2019 Cochrane risk of bias tool 2.0
Models and methods of meta-analysis	Random effects model; inverse variance method.	Random effects model; no method described.	Mantel-Haenszel fixed effect model primarily; random effects models when statistical heterogeneity was observed.	Random effects model; no method described.	Fixed effect model; random effects model when there was heterogeneity. No method described.	Random effects model; inverse variance method.
Definition of heterogeneity	Cochran's Q test $p < 0.1$. I^2 statistic (<25%, 25 to 50%, >50% were modest, moderate and substantial, respectively).	I^2 statistic (<25%, 25 to 50%, >50% were low, moderate and high, respectively).	Chi^2 test $p < 0.05$; I^2 statistic (25%, 50%, and 75% were low, medium, and high levels of heterogeneity, respectively).	Cochran's Q and I^2 index, (I^2 >50% defined as significant heterogeneity).	Q-statistic; amount of heterogeneity with I^2 statistic. Heterogeneity of any kind was defined as I^2 >0%.	I^2 statistic (<30%, 30-60%, and >60% were low, medium, and high, respectively).
Blood pressure or other continuous outcome association measure: Effect (95%CI)	MD (control minus RDN): *24-h SBP*: -2.81 (-6.46 to 0.83) *Office SBP*: -4.89 (-20.9 to 11.1) *eGFR*: 0.81 ml/min/1.73m^2 (-1.69 to 3.3)	MD (RDN minus control): *24-h ambulatory SBP:* -8.23 (-16.86 to 0.39) *24-h ambulatory DBP*: -3.77 (-7.21 to -0.32) *Office SBP*: -8.23 (-16.86 to 0.39) *Office DBP*: -3.77 (-7.21 to -0.32)	MD (RDN minus control): *24-h ambulatory SBP*: 0.28 (3.74 to 4.29) *24-h ambulatory DBP*: 0.93 (-4.50 to 6.36) *Office SBP*: -4.08 (-15.26 to 7.11) *Office DBP*: -1.30 (-7.30 to 4.69) *Serum creatinine*: 0.01 mg/dL (-0.12 to 0.14) *CrCl*: -2.09 mL/min (-8.12 to 3.95)	MD (RDN minus control): *24-h ambulatory SBP*: -3.45 (-5.01 to -1.88) *24-h ambulatory DBP*: -1.56 (-2.81 to -0.30) *Office SBP*: -3.99 (-8.10 to 0.11) *Office DBP*: -2.97 (-4.76 to -1.18)	MD (RDN minus control): *24-h ambulatory SBP*: -4.02 (-5.49 to -2.56) *Office SBP*: -8.93 (-14.03 to -3.83)	MD (RDN intervention vs RDN intervention) in NMA<br><br>RF in MRA and branches vs: *24-hour ambulatory SBP*: RF in MRA -7.8 (-15.1 to -0.4), RF in MRA plus AHT -11.9 (-23.4 to -0.4), sham -7.2 (-13.6 to -0.8), and AHT -12.9 (-22.6 to -3.2) *24-hour ambulatory DBP*: RF in MRA -4.2 (-8.3 to -0.2), sham -3.7 (-7.1 to -0.2), and AHT -6.8 (-12.7 to -0.8)

						Nighttime SBP: RF in MRA -7.6 (-14.6 to -0.7)<br><br>RF in MRA plus AHT vs: *Office SBP*: AHT **-10.1 (-21.4 to -0.6)**<br>*Office DBP*: AHT -5.4 (-9.6 to -1.1)<br><br>Other effects were not significant.
Clinical outcomes association measure:<br>Effect (95%CI)	Not assessed	Not assessed	RR:<br>*MI*: 1.31 (0.45 to 3.84)<br>*Ischaemic stroke*: 1.15 (0.36 to 3.72)<br>*Unstable angina*: 0.63 (0.08 to 5.06)<br>*Bradycardia episodes*: 6.63 (1.19 to 36.84)<br><br>Fatal and non-fatal CV events, all-cause mortality, hospital admissions, and quality of life without effects due to scarce information.	Not assessed	RR:<br>*Major adverse events*: 1.06 (0.72 to 1.57)<br><br>Major adverse events defined as all-cause mortality, vascular complications (acute coronary event, cerebrovascular event or renal artery complications), renal complications, hypertensive crisis and heart failure.	Scarce data.<br>No significant differences between MRA +/- branches or MRA +AHT and Sham or AHT for heart failure, stroke, MI, renal complications, hypertensive crisis, and serious adverse events. Other outcomes (overall mortality, CV mortality, and hospitalization of any cause) did not have data to analyze.

Ranking of best RDN interventions	Not assessed	Not assessed	Not assessed	Not assessed	Not assessed	RF MRA and branches: best intervention for 24h ambulatory, daytime, and nighttime SBP and DBP (p scores from 0.83 to 0.97)<br>RF MRA plus AHT: best intervention for office SBP and DBP (p scores 0.84 to 0.90). No NMA possible for clinical outcomes due to scarcity of data.
Conclusion	In RH, RDN with the Symplicity system did not significantly decrease BP but was safe.	RF RDN did not have superiority compared with medical treatment at 6-month follow-up in general population.	Low quality evidence that RDN did not change major CV events and renal function. Moderate quality evidence that RDN did not change BP. Low quality evidence that RDN increased bradycardia events.	RDN reduces ambulatory BP and office DBP in patients with hypertension.	Catheter-based RDN was associated with a significant BP lowering benefit without increasing major adverse events.	RF in MRA and branches was the most efficacious in comparison to other interventions to treat RH or UH. Clinical and adverse events were uncommonly described in existing trials.

All blood pressures measured as mmHg. HTN: hypertension; US: Ultrasound; MRA: Main renal artery; RDN: Renal denervation; BP: blood pressure; SBP: Systolic blood pressure; DBP: Diastolic blood pressure; AHT: Antihypertensive; RF: radiofrequency; ABPM: Ambulatory blood pressure monitoring; RH: resistant hypertension; UH: uncontrolled hypertension; RCT: randomized controlled trials; CI: confidence interval; MD: mean difference; SMD: standardized mean difference; RR: risk ratio; MI: Myocardial infarction; CV: cardiovascular.

Figure S1. Network geometries for primary and secondary outcomes.

A. 24h Ambulatory SBP

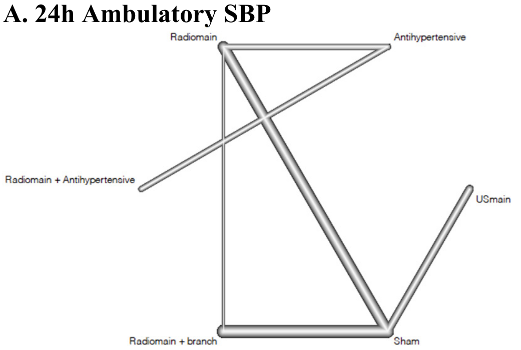

B. Office SBP

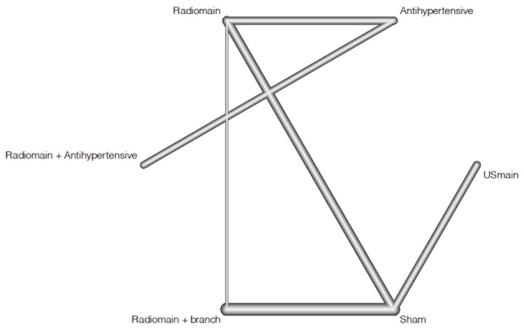

C. 24h Ambulatory DBP

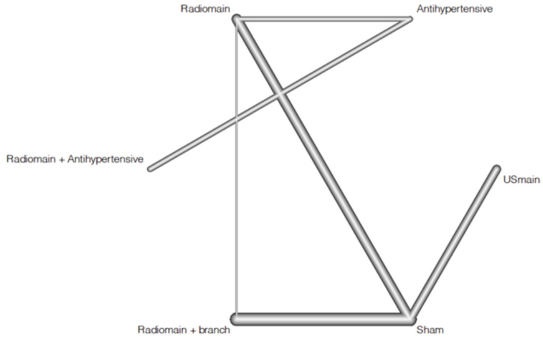

D. Office DBP

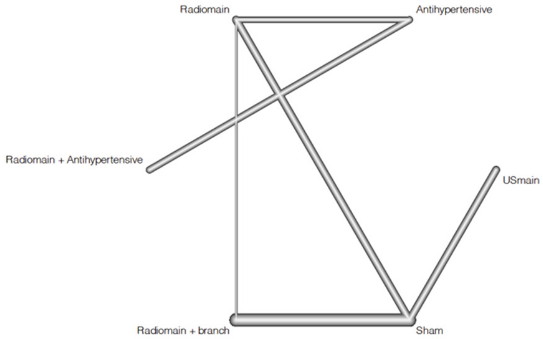

E. Daytime SBP

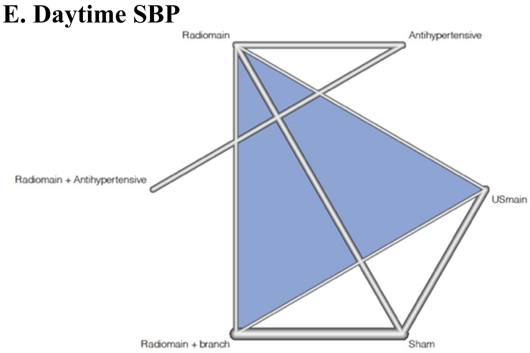

F. Nighttime SBP

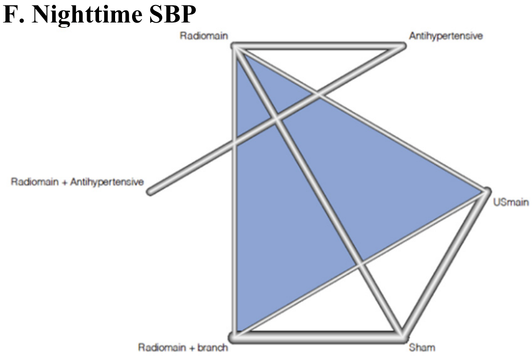

G. Daytime DBP

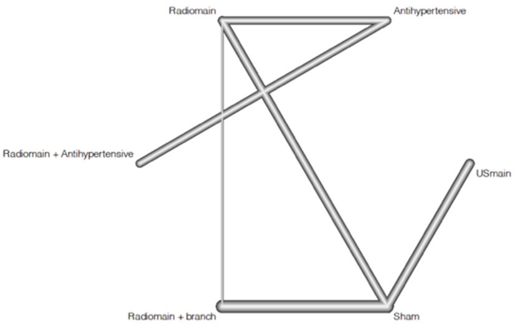

H. Nighttime DBP

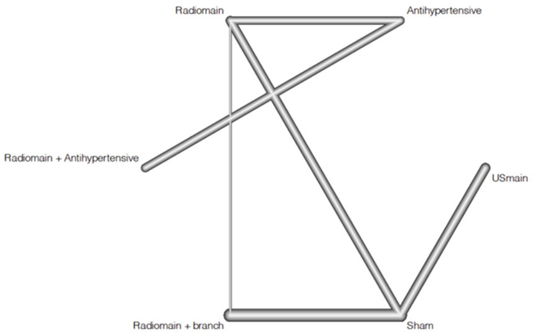

Figure S2. Risk of bias per domain of included randomized trials.

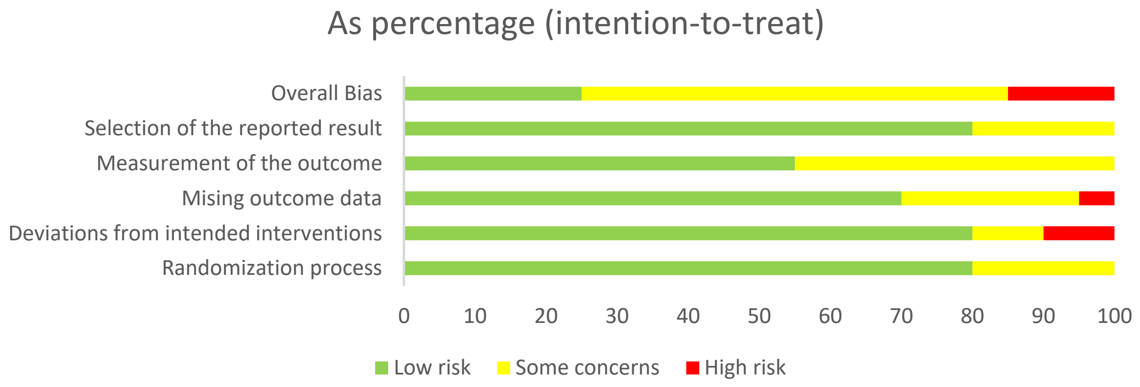

Figure S3. Effect of renal denervation interventions on change of 24h ambulatory SBP in comparison to antihypertensive drugs.

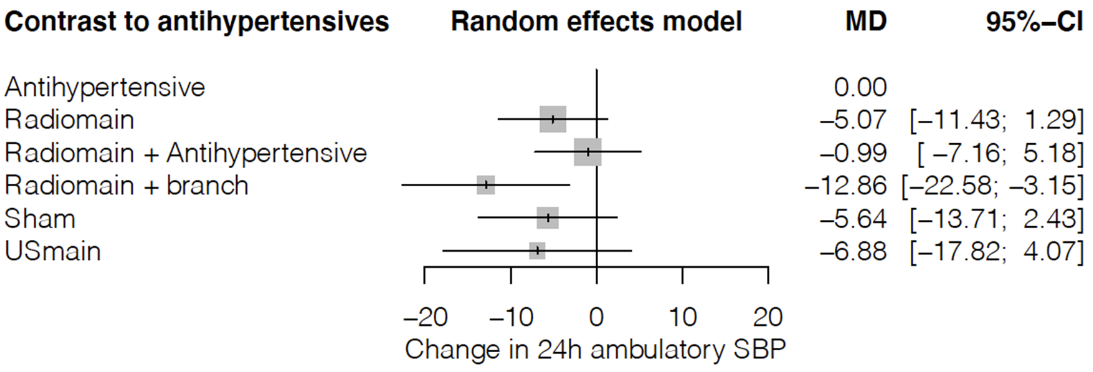

Figure S4. Effect of renal denervation interventions on change of office SBP in comparison to antihypertensive drugs.

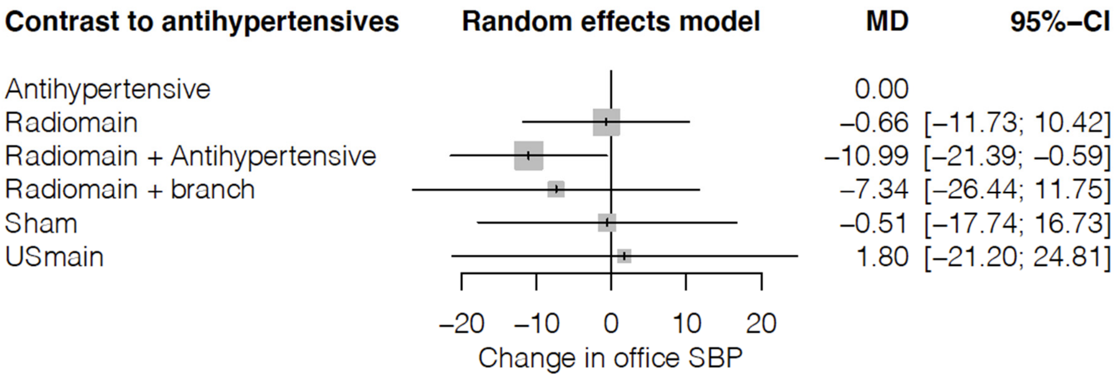

Figure S5. Effect of renal denervation interventions on change of 24h ambulatory DBP in comparison to antihypertensive drugs.

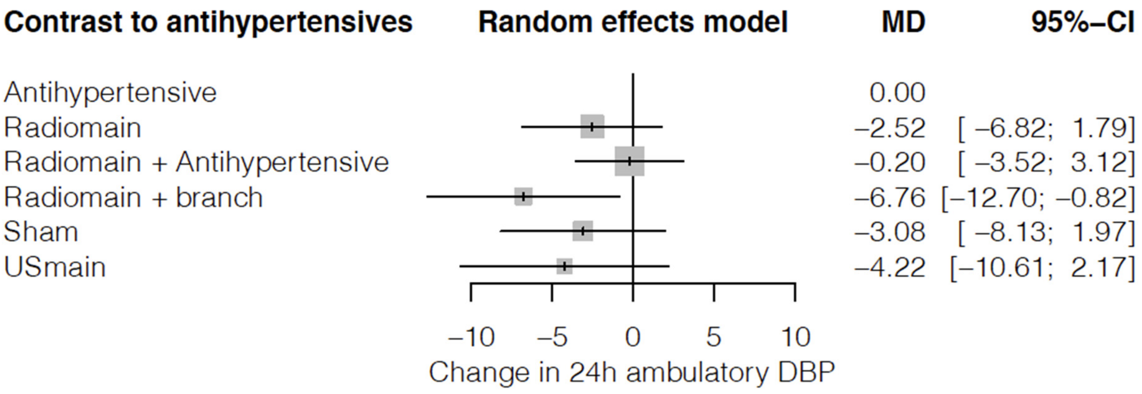

Figure S6. Effect of renal denervation interventions on change of office DBP in comparison to antihypertensive drugs.

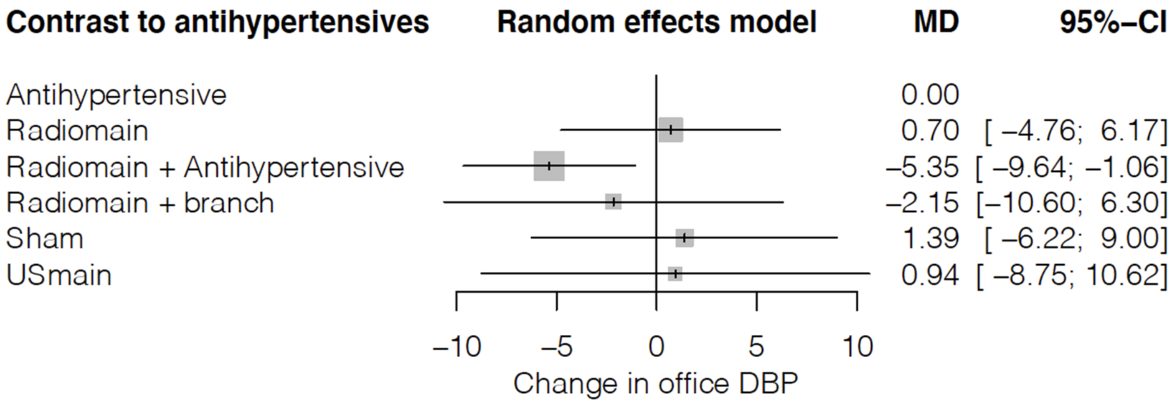

Figure S7. Effect of renal denervation interventions on change of daytime SBP in comparison to antihypertensive drugs.

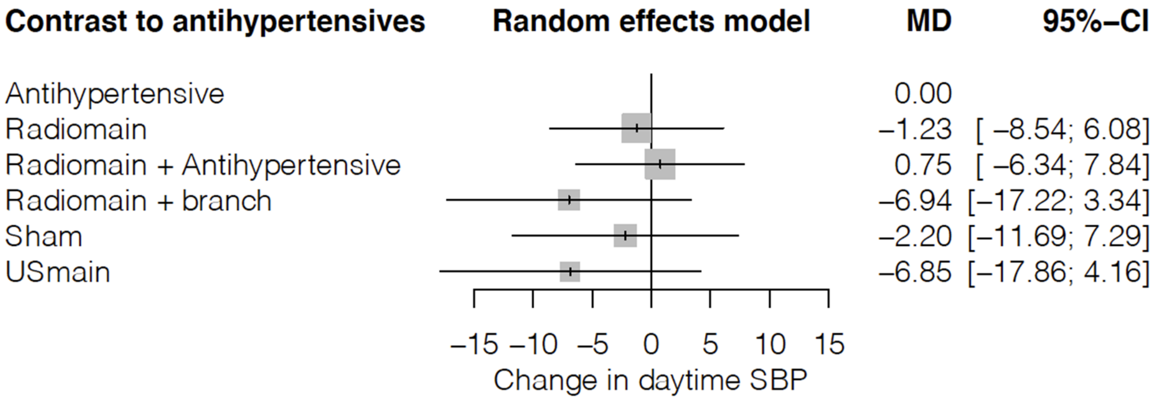

Figure S8. Effect of renal denervation interventions on change of nighttime SBP in comparison to antihypertensive drugs.

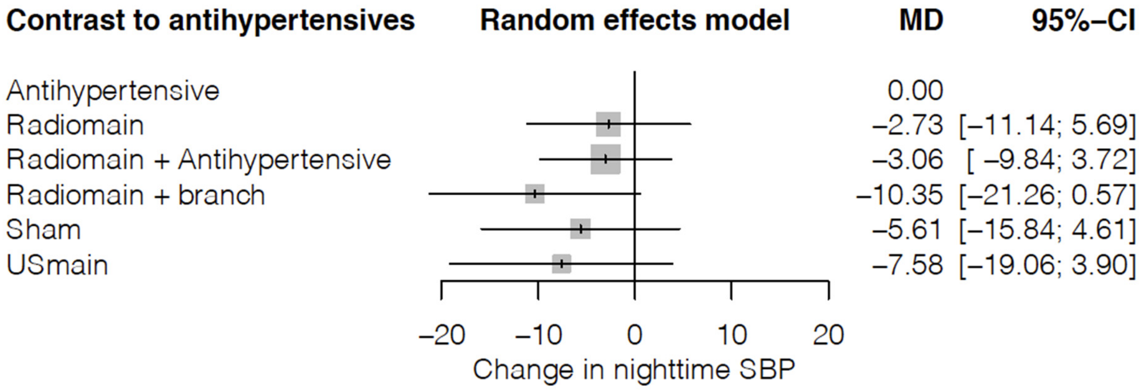

Figure S9. Effect of renal denervation interventions on change of daytime DBP in comparison to antihypertensive drugs.

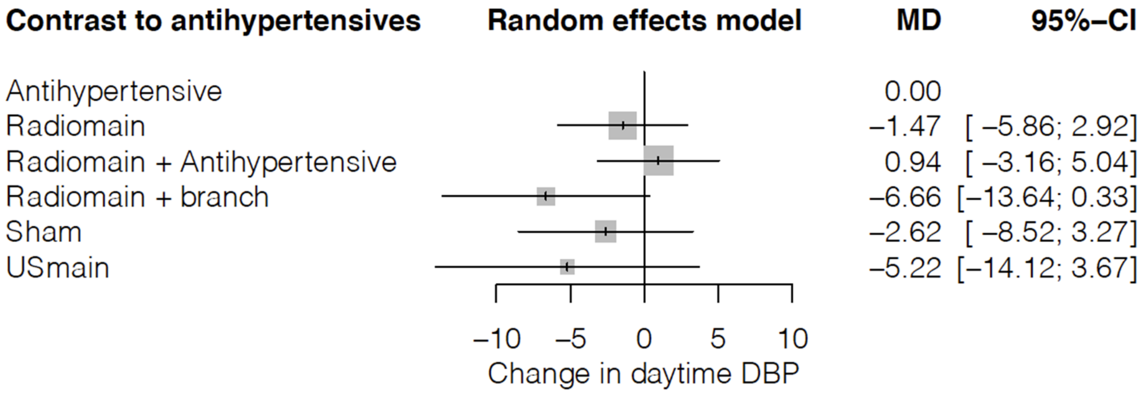

Figure S10. Effect of renal denervation interventions on change of nighttime DBP in comparison to antihypertensive drugs.

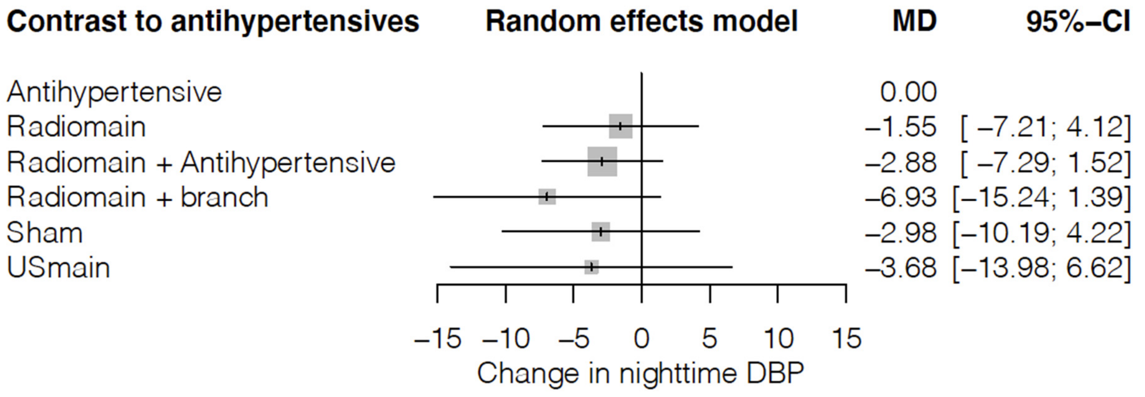

Figure S11. Effect of RF MRA vs sham on heart failure.

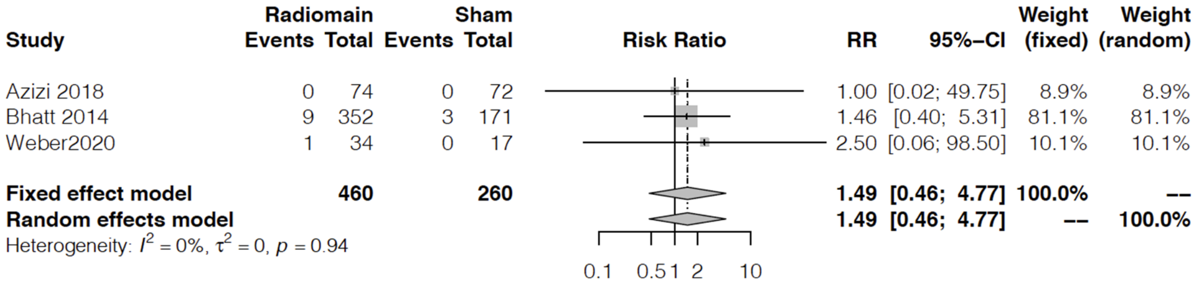

Figure S12. Effect of RF MRA vs sham on stroke.

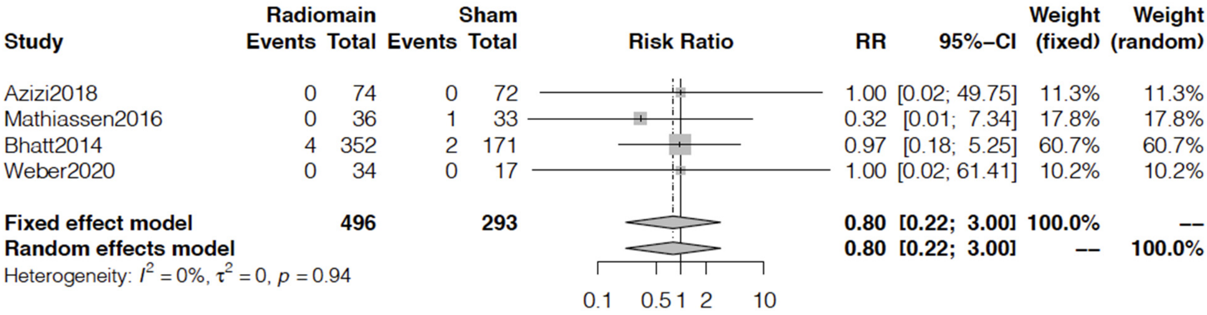

Figure S13. Effect of RF MRA + branch vs sham on stroke.

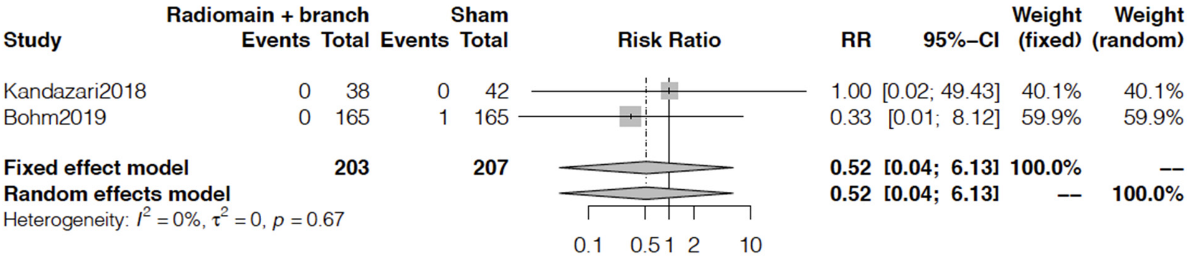

Figure S14. Effect of RF MRA +/- branch vs sham on stroke.

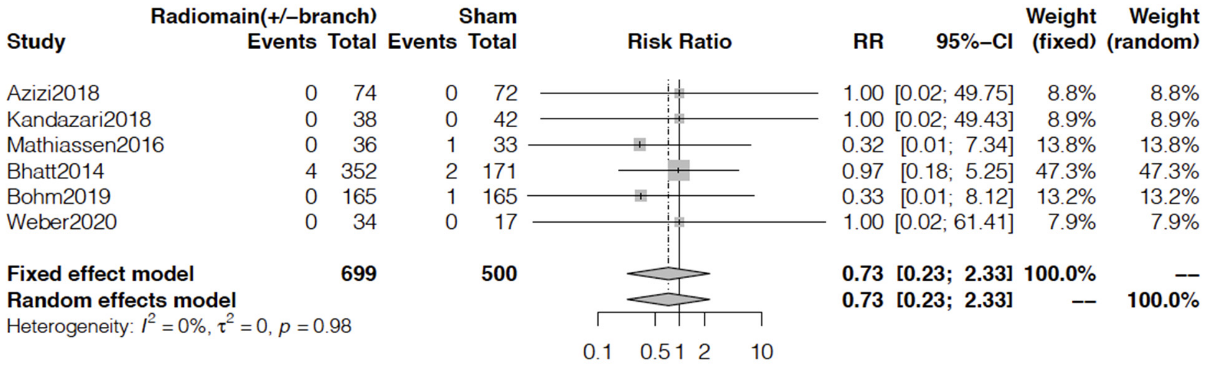

Figure S15. Effect of RF MRA vs sham on myocardial infarction.

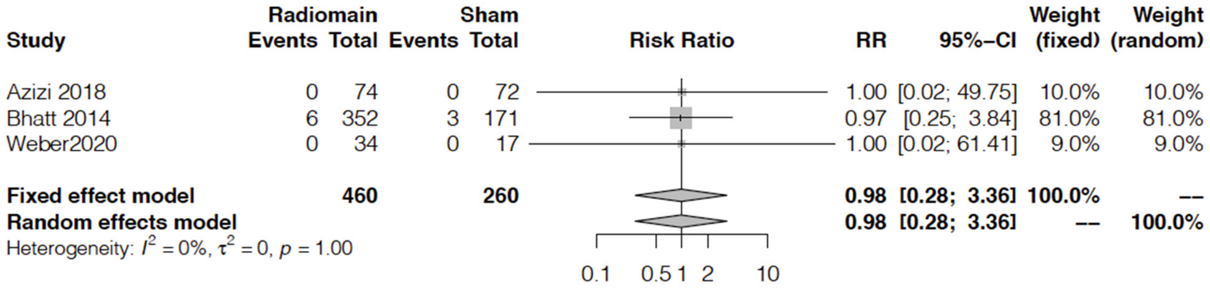

Figure S16. Effect of RF MRA + branch vs sham on myocardial infarction.

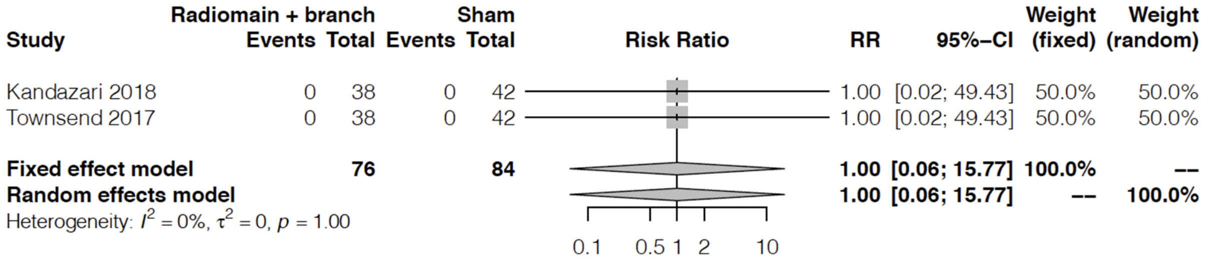

Figure S17. Effect of RF MRA +/- branch vs sham on myocardial infarction.

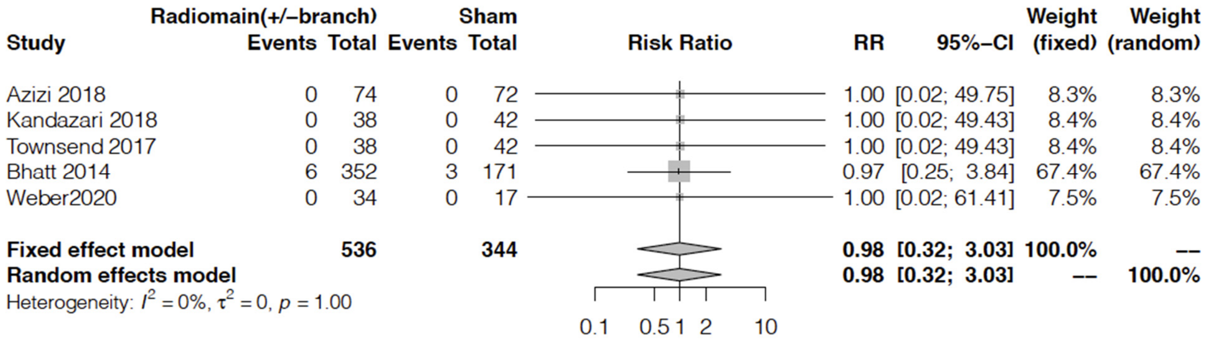

Figure S18. Effect of RF MRA vs sham on renal complications.

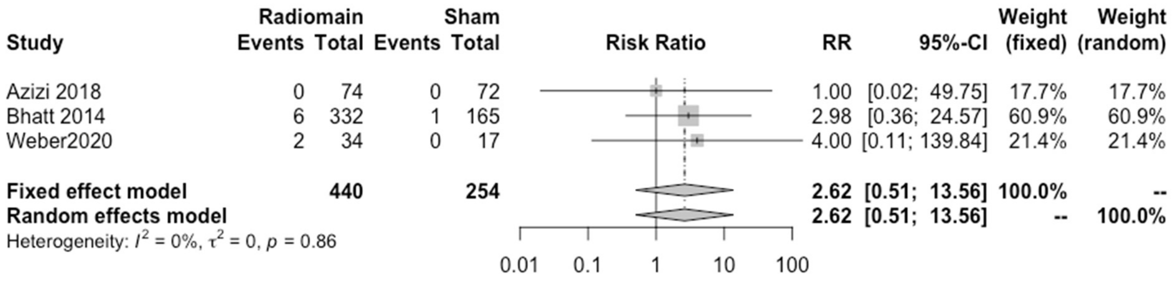

Figure S19. Effect of RF MRA + branch vs sham on renal complications.

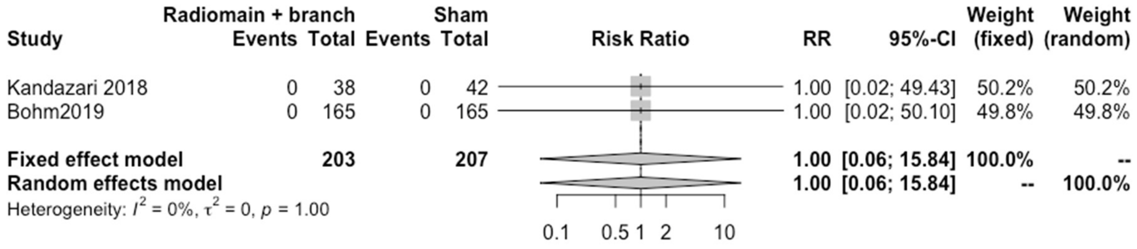

Figure S20. Effect of RF MRA +/- branch vs sham on renal complications.

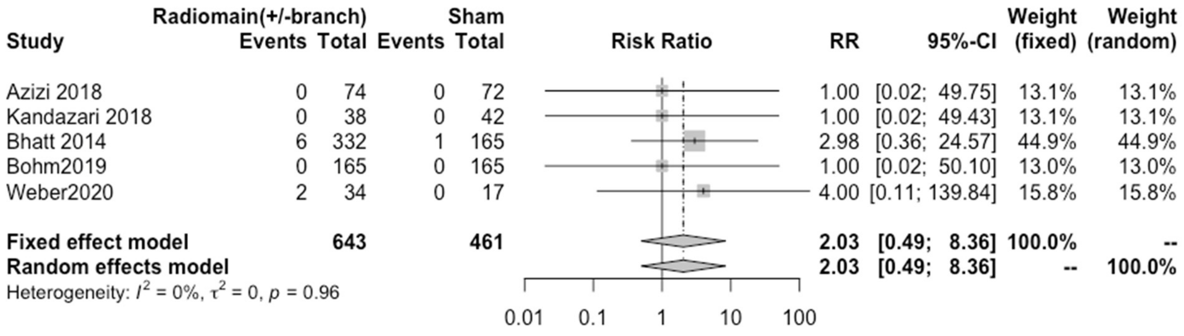

Figure S21. Effect of RF MRA vs sham on hypertensive crisis.

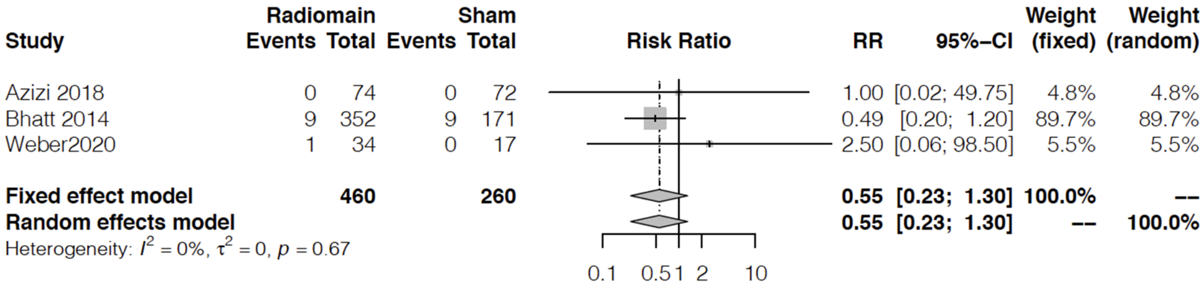

Figure S22. Effect of RF MRA + branch vs sham on hypertensive crisis.

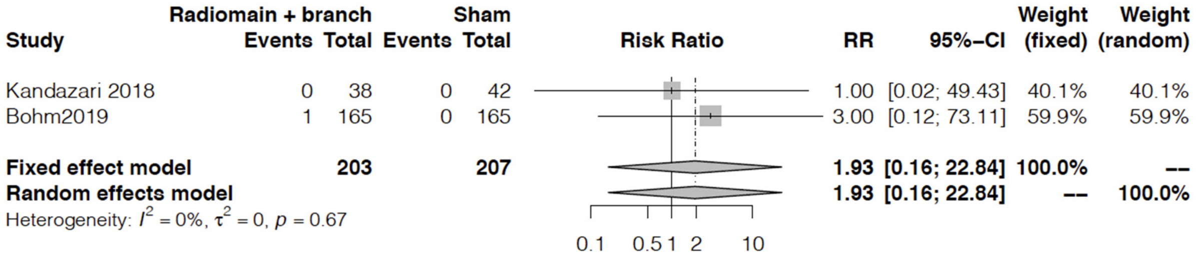

Figure S23. Effect of RF MRA +/- branch vs sham on hypertensive crisis.

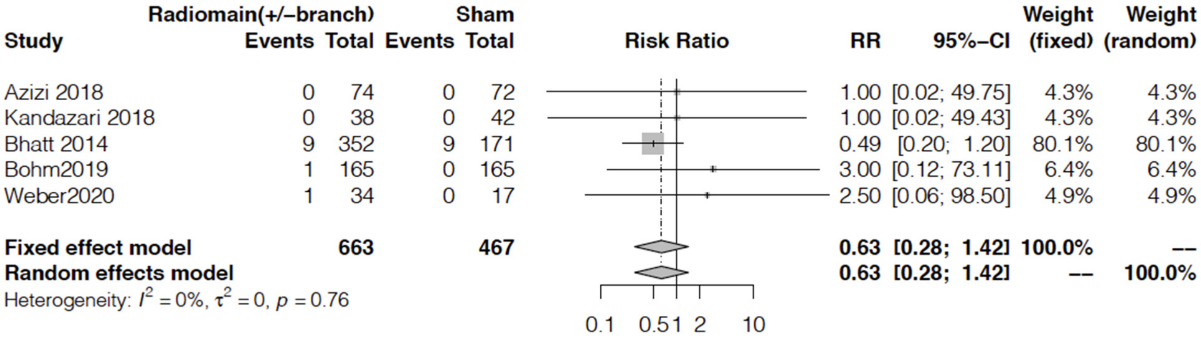

Figure S24. Effect of RF MRA vs sham on serious adverse events.

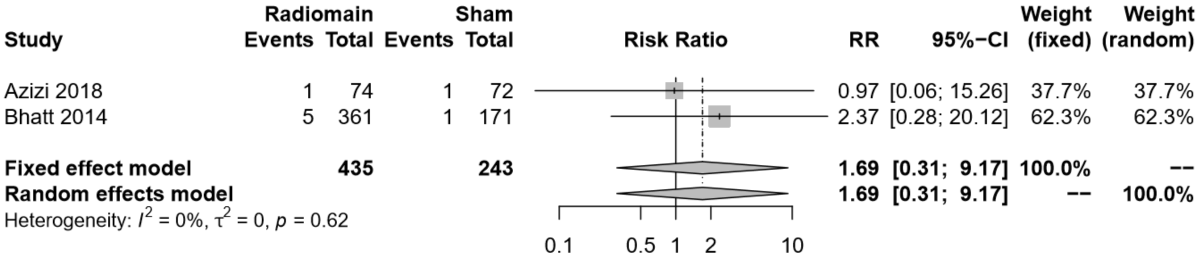

Figure S25. Effect of RF MRA + AHT vs AHT on serious adverse events.

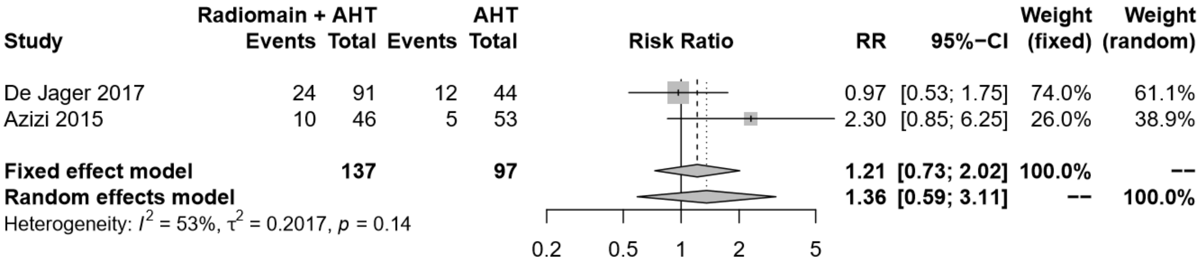

Figure S26. Effect of RF MRA +/- branch vs sham on serious adverse events.

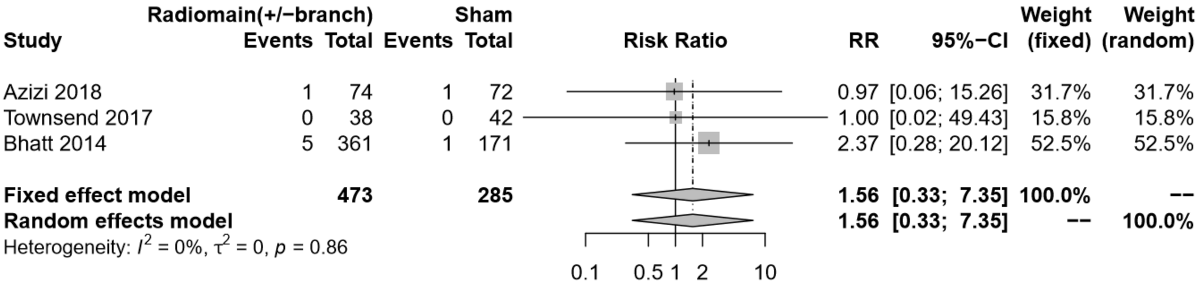

Supplement: Supplementary file 1 [file jcm-10-00782-s001.pdf]
